# Distinct spatial distribution of potentiated dendritic spines in encoding- and recall-activated hippocampal neurons

**DOI:** 10.3389/fnmol.2025.1751677

**Published:** 2026-01-20

**Authors:** Francesco Gobbo, Ajesh Jacob, Bruno Pinto, Marco Mainardi, Laura Cancedda, Antonino Cattaneo

**Affiliations:** 1 Bio@SNS, Scuola Normale Superiore, Pisa, Italy; 2 Centre for Discovery Brain Sciences and UK Dementia Research Institute, The University of Edinburgh, Edinburgh, United Kingdom; 3 Brain Development and Disease Laboratory, Istituto Italiano di Tecnologia (IIT), Genova, Italy; 4 Department of Biomedical Sciences, University of Padova, Padova, Italy; 5 Telethon Dulbecco Institute, Rome, Italy; 6 Rita Levi-Montalcini European Brain Research Institute (EBRI), Rome, Italy

**Keywords:** dendritic spine, engram, fear conditioning, hippocampus, long-term potentiation, memory, synaptic plasticity, dendritic engram

## Abstract

Experimental advancements in neuroscience have identified cellular engrams—ensembles of neurons whose activation is necessary and sufficient for memory retrieval. Synaptic plasticity, including long-term potentiation, is fundamental to memory encoding and recall, but the relationship between learning-induced dendritic spine potentiation and neuron-wide activation remains unclear. In this study, we employed a post-synaptic translation-dependent reporter consistent with potentiation (SA-PSDΔVenus) and a neuronal activation reporter (ESARE-dTurquoise) to determine their spatiotemporal correlation in the mouse hippocampal CA1 following contextual fear conditioning (CFC). SA-PSDΔVenus+ spines were enriched in ESARE-dTurquoise+ neurons, with distribution varying across CA1 layers at different phases of memory: SA-PSDΔVenus+ were more frequent in activated neurons in *stratum oriens* and *stratum lacunosum moleculare* after CFC (encoding), while recall-activated neurons showed a larger number of SA-PSDΔVenus+ in the *stratum radiatum*. These findings demonstrate that the relative weight and spatial distribution of potentiated synaptic inputs to hippocampal CA1 pyramidal neurons change between the encoding and retrieval phases of memory.

## Introduction

The search for the physical substrate of memory has been a long-standing focus of extensive research (Josselyn et al., 2017). In recent decades, the development of novel genetic tools for identifying and manipulating neurons activated during learning has provided new insights into the formation, storage, and retrieval of memories (Poo et al., 2016). A majority of these approaches rely on immediate early genes (IEGs), such as *c-fos, Arc*, and *Zif268*, which are rapidly and transiently expressed following neuronal activation (Guzowski, 2002; Minatohara et al., 2016). Using IEG promoters to drive reporter proteins or optogenetic and chemogenetic actuators, neurons activated during learning have been identified across multiple brain regions, including the hippocampus, the amygdala, and the cortex (Reijmers et al., 2007; Garner et al., 2012; Liu et al., 2012; Denny et al., 2014; Vetere et al., 2017). Artificial reactivation of these neurons can elicit behavioral responses consistent with memory retrieval, whereas their inhibition impairs memory expression, supporting the concept of cellular engrams (Liu et al., 2012; Denny et al., 2014; Josselyn and Tonegawa, 2020).

Bidirectional modifications of synaptic strength, collectively termed synaptic plasticity, have long been considered the neural correlate of learning (Andersen et al., 2007; Takeuchi et al., 2014; Bliss et al., 2018). Learning-induced increases in synaptic strength have been reported in the hippocampus and amygdala (Rogan et al., 1997; Whitlock et al., 2006; Pavlowsky et al., 2017). Pharmacological or genetic disruption of synaptic plasticity impairs adaptive behaviors in response to natural retrieval cues, although whether these interventions interfere with memory acquisition (or learning) or recall is unclear (Morris et al., 1986; Tsien et al., 1996; Plath et al., 2006). Moreover, inducing long-term depression (LTD) and long-term potentiation (LTP) in the neuronal network modified during learning impairs and reactivates memory, respectively, thus supporting a causal association between synaptic plasticity and memory (Nabavi et al., 2014).

Despite these advances, the relationship between synaptic plasticity and the ensemble(s) of neurons activated by learning is still unclear. Synaptic plasticity, detected as an increase in spine density and the ratio of a-amino-3-hydroxy-5-methyl-4-isoxazolepropionic acid (AMPA) to N-methyl-D-aspartate (NMDA) receptor currents, has been observed in neurons activated during learning (Ryan et al., 2015; Kitamura et al., 2017; Choi et al., 2018, 2021). Disrupting synaptic plasticity within these neurons impairs their reactivation and memory recall (Ryan et al., 2015; Abdou et al., 2018). The reactivation probability of learning-activated neurons changes over time and correlates with dynamic alterations in spine density, suggesting ongoing synaptic remodeling (Kitamura et al., 2017; Tonegawa et al., 2018).

Theoretical models and experimental studies suggest that individual memories maintain segregated synaptic representations even when they largely overlap at the cellular level (Kastellakis et al., 2016; Abdou et al., 2018). However, since individual neurons contain tens of thousands of synapses, it remains unclear which specific subsets of synapses are modified during learning and contribute to memory storage (Megias et al., 2001; Poo et al., 2016; Gobbo and Cattaneo, 2020). To address this fundamental question, novel genetic tools are being engineered to identify and manipulate the synaptic correlate of a memory (Makino and Malinow, 2011; Hayashi-Takagi et al., 2015; Gobbo et al., 2017; Dore et al., 2020; Perez-Alvarez et al., 2020; Kim et al., 2025). We previously developed SynActive, a strategy that drives local, translation-dependent expression of reporter proteins specifically at potentiated dendritic spines using regulatory sequences from *Arc* mRNA and spine-targeting peptides (Gobbo et al., 2017).

In this study, we use SynActive to assess layer-specific and time-dependent patterns of learning-induced potentiated dendritic spines in hippocampal CA1 neurons following contextual fear conditioning (CFC). By combining SynActive with a neuronal activation reporter, we examined how potentiated spines are distributed between active and inactive neurons during memory encoding and recall. Our results reveal a positive correlation between learning-induced synaptic potentiation and neuronal activation, with the strength of this relationship varying across CA1 layers and time points after CFC.

## Materials and methods

### Animals

Embryonic day (E) 15.5 timed-pregnant CD1 mice (Charles River SRL, Italy) were used for the *in vivo* experiments. Time-pregnant matings were performed in the evening; the day after mating was defined as E0.5, and the day of birth was defined as P0. Mice were kept under a 12-h dark-to-light cycle, with *ad libitum* access to food and water. Mice from both sexes were P26 on the day of the experiment and were assigned blindly between groups. Animal care and experimental procedures were approved by the Italian Institute of Technology licensing and the Italian Ministry of Health. Primary hippocampal neurons were prepared from P0 B6126 mice. All animal procedures were approved by the Italian Ministry of Health and the Italian National Research Council (CNR).

### Plasmids

To minimize overexpression artifacts while maintaining its localization to dendritic spines, PDZ1 and PDZ2 domains were deleted from the PSD95 construct (PSDΔ; rat PSD95; NCBI ID NM_019621.2, nucleotides 57–248 and 993–2228; Arnold and Clapham, 1999; Hayashi-Takagi et al., 2015). PSDΔVenus was obtained by joining two PCR fragments amplified from FU-dio PSD95-mCherry-W (Addgene 73919) and cloning in fusion with fluorescent protein mVenus (Nagai et al., 2002; see also Supplementary Table 1). pCMV-PSDΔVenus was then generated by inserting PSDΔVenus into pcDNA3.1(+) using NheI/XbaI restriction sites. pCMV-SA-PSDΔVenus was generated analogously by inserting PSDΔVenus between the 5′ and 3′ *Arc* UTRs in pcDNA3.1(+) using NheI/XhoI sites as previously described in the study of Gobbo et al. (2017). 5′ and 3′ *Arc* UTR comprises nucleotides 1–230 and 1424–3026 of rat Arc transcript (NCBI NM_019361.2; see also Supplementary Table 1).

To generate pTRE3-SA-PSDΔVenus-HA-CK-rtTA, the cDNA for the haemagglutinin (HA) tag was first inserted in-frame at the end of the PSDΔVenus cDNA in pCMV-SA-PSDΔVenus. The resulting cDNA, the SA-PSDΔVenus-HA cDNA, was amplified by PCR containing the *Arc* UTR sequences and cloned into pTRE3-SA-CK-rtTA (Gobbo et al., 2017) after the removal of the previous coding sequence. The resulting vector expresses SA-PSDΔVenus (PSDΔVenus-HA flanked by 5′ and 3′ *Arc* UTRs) from the third-generation tetracycline-sensitive promoter (Sato et al., 2013) and the rtTA2S-M2 transactivator from the minimal CamKII(0.4) promoter. To generate pAAV-TRE3-SA-PSDΔVenus, TRE3 and SA-PSDΔVenus were cloned into pAAV-hSyn-EGFP (Addgene 50465) by replacing the sequences between the inverted terminal repeats (ITRs).

pCAGGS-TdTomato (Szczurkowska et al., 2016) expresses TdTomato from CAGGS promoter and pCAGGS-rtTA-IRES-mCherry (Gobbo et al., 2017), express tetracycline/doxycycline sensitive TetON transcription factor (rtTA) and TdTomato. pAAV-hSyn-rtTA-P2A-tdTomato was generated by cloning rtTA-P2A (P2A sequence in the 3′ end) and tdTomato between the hSyn promoter and WPRE of pAAV-hSyn-EGFP (Addgene 50465).

pESARE-dTurquoise was generated from the pCAGGS backbone after removing the CAGGS-IRES-TdTomato cassette (thus leaving the polyA site) and inserting the cDNA encoding the activity-dependent promoter E-SARE (Kawashima et al., 2013) and the fusion protein FLAG-mTurquoise2-d2. The E-SARE sequence contains five copies of the SARE enhancer and was generated as described previously in the study by Kawashima et al. (2013). To generate the FLAG-mTurquoise2-d2, the cDNA encoding mTurquoise2 was amplified from pPalmitoyl-mTurquoise2 (Addgene 36209) and inserted into the cDNA for the 3xFLAG tag (*N-*DYKDHDGDYKDHDIDYKDDDDK*-C*) at the 5′ end and the cDNA encoding the destabilization sequence (*N-*HGFPPEVEEQDDGTLPMSCAQESGMDRH*-C*) from mouse ornithine decarboxylase (Li et al., 1998) at the 3′ end.

### Cell culture experiments

Primary hippocampal neurons were prepared from P0 B6126 mice as previously described by Gobbo et al. (2017; see Supplementary Methods for details). On div 10, neurons were transfected with pCMV-SA-PSDΔVenus/pCAGGS-TdTomato or pCMV-PSDΔVenus/pCAGGS-TdTomato with the calcium phosphate method (see Supplementary Methods for details). The following day, neurons were treated with 10 mM KCl (Sigma Aldrich P9333) for 90 min or equivalent volume in saline. A third group was incubated with 100 μM AP5 (Tocris 0106) overnight from the end of transfection. Neurons were then fixed in 2% PFA (Sigma Aldrich P6148), 0.5% sucrose (Sigma Aldrich S7903) in phosphate-buffered saline (PBS) for 10 min, and then washed and maintained in PBS. Neurons were imaged using a confocal microscope (see Supplementary Methods for details).

For the colocalization analysis of SA-PSDΔVenus and GluA1, div 3 neurons were transfected with pAAV-TRE3-SA-PSDΔVenus/pAAV-hSyn-rtTA-P2A-tdTomato using Lipofectamine 2000 (Thermo Fisher 11668019) according to the manufacturer's instructions (see Supplementary Methods for details). Doxycycline (Sigma-Aldrich D9891, final concentration 1 μg/ml) was added to cultures the evening before the induction of Glycine-mediated chemical LTP (Gly-cLTP), between div 14–16. Gly-cLTP was induced in cultured hippocampal neurons, as previously described by (Lu et al. 2001; see Supplementary Methods for details). Ninety minutes post Gly-cLTP, neurons were fixed in 4% PFA, immunolabeled with GluA1, and imaged using a confocal microscope (see Supplementary Methods for details).

### *In utero* electroporation and animal experiments

Hippocampal *in utero* electroporation was performed as previously described by Szczurkowska et al. (2016). Embryonic day (E) 15.5 timed-pregnant CD1 mice (Charles River SRL, Italy) were used. Time-pregnant matings were performed in the evening; the day after mating was defined as E0.5, and the day of birth was defined as P0. Embryos from time-pregnant mothers were electroporated unilaterally with pTRE3-SA-PSDΔVenus-HA-CK-rtTA/pCAGGS-rtTA-IRES-mCherry/pESARE-dTurquoise. Mice from both sexes were P26 on the day of the experiment and were assigned blindly between groups. Animals received 0.5 mg doxycycline (1 mg/30 g body weight) in saline solution intraperitoneally the evening before the experiment and early in the morning of the experiment. After 3–4 h, animals were put in the fear chamber (a square box with metal rods on the floor). After 3 min in the chamber, a 2-s 0.75 A shock was administered through the metal floor and maintained in the chamber for 30 s after the shock. The CFC group was fixed by transcardial perfusion with 4% formaldehyde in phosphate-buffered saline (PBS), 90 min after exiting the chamber. Due to the time necessary for animal preparation and perfusion, up to 120 min could pass between the end of conditioning and the fixation of the brain. In the study, this time interval will be considered as 90 min. Mice from the HC group were injected identically but were maintained in their home cage and were not exposed to CFC. HC animals were perfused at times matched with the CFC group. Animals from the AA and AB groups were injected and conditioned as the CFC group but were returned to their home cage after CFC until the following day. Twenty-four hours after CFC, animals from the AA group were put in the conditioned chamber for 3 min with no shock, while the AB animals were put in the control chamber (a square box with a different floor and walls) for the same amount of time. Both groups were perfused after 90 min. The HC24 and CFC24 groups were injected and treated as the HC and CFC groups, respectively, but were perfused 24 h after CFC (or equivalent time for HC24 animals) instead of 90 min. Perfused brains were sliced, and the hippocampal region was imaged using a confocal microscope (see Supplementary Methods for details).

### Quantification and statistical analysis

Image analysis was performed using ImageJ (Schindelin et al., 2012; see Supplementary Methods for details). Statistical analysis was performed with OriginPro v9.0 or GraphPad Prism 8. For comparing SA-PSDΔVenus and PSDΔVenus expression in primary neuronal cultures, a two-way ANOVA followed by Holm-Sidak's multiple comparison test was performed. Paired Student's *t*-test was used to compare the a-amino-3-hydroxy-5-methyl-4-isoxazolepropionic acid (AMPA) receptor enrichment in primary neuronal cultures. The number of SA-PSDΔVenus+ spines in the CA1 hippocampal neurons under different experimental conditions was analyzed using a one-way ANOVA, followed by Tukey's multiple comparison test, or a two-way ANOVA, followed by Bonferroni's multiple comparison test.

Statistical details and sample sizes can also be found in each Figure legend, and the minimum significance level defined was a *p*-value of <0.05. The bar graphs in the images show mean ± SEM.

## Results

### SA-PSDΔVenus is expressed at potentiated spines in primary hippocampal neurons

**Figure 1:**
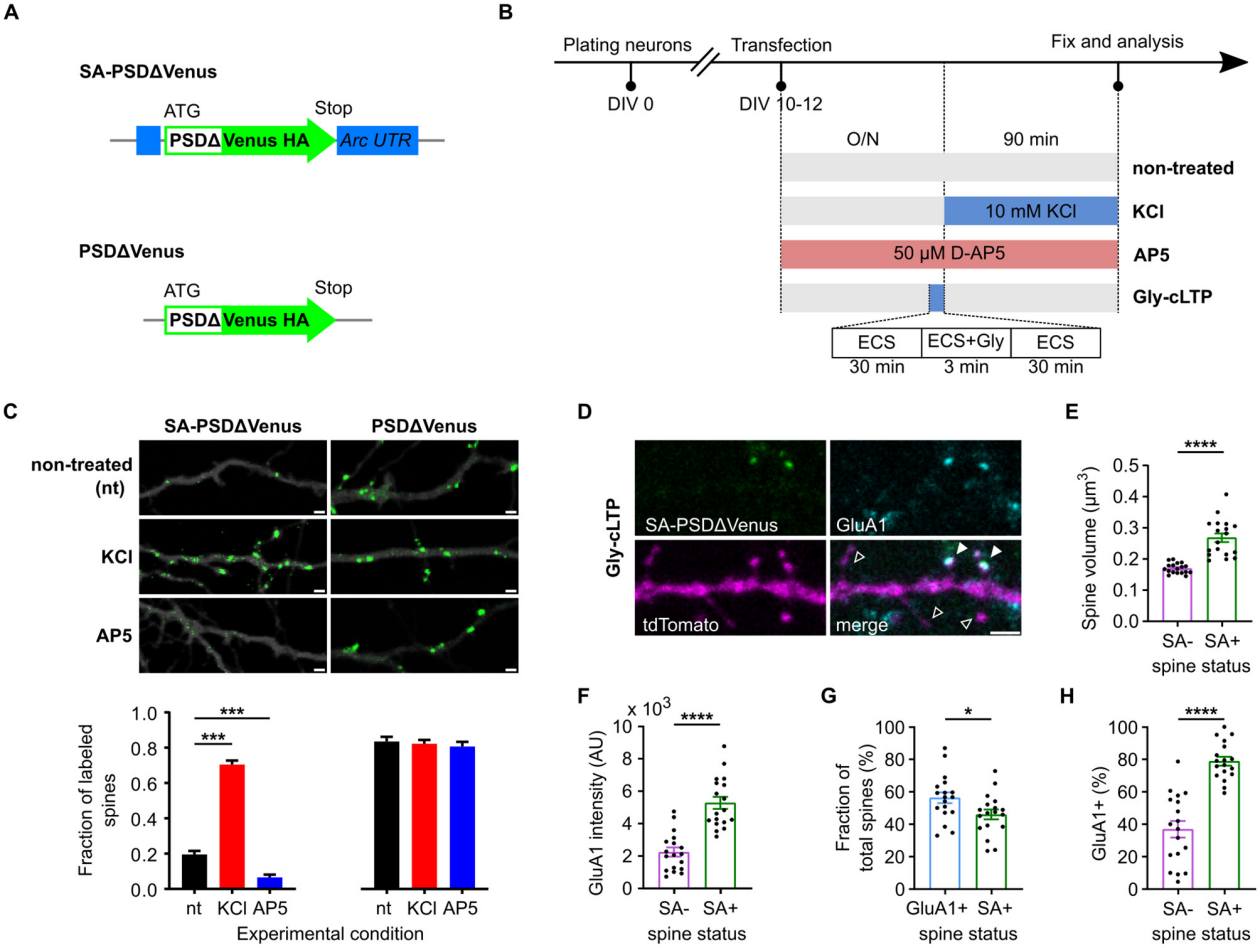
SA-PSDΔVenus is expressed at potentiated spines in an activity- and NMDA receptor-dependent manner. **(A)** Schema of the SA-PSDΔVenus construct and the control PSDΔVenus construct. **(B)** Schematic of the treatments used to modulate synaptic activity in the primary neuron cultures. **(C)** Primary mouse hippocampal neurons expressing tdTomato (greyscale) and SA-PSDΔVenus or PSDΔVenus (green) after 24 h D-AP5 (AP5), 90 min KCl (KCl) or non-treated (nt) (upper). Scale bar, 1 μm. Bar plot shows the fraction of SA-PSDΔVenus+ or PSDΔVenus+ spines in each condition (lower). ***P < 0.001, two-way ANOVA, followed by Holm-Sidak's multiple comparison test; interaction *F*_(2, 108)_ = 90.96, *P* < 0.001. **(D)** Representative image of a dendritic segment from cultured hippocampal neurons after Gly-cLTP treatment, expressing SA-PSDΔVenus (green) and tdTomato (magenta), and immunolabeled for the AMPAR subunit GluA1 (cyan). The merged image shows all three channels. Filled arrowheads indicate SA-PSDΔVenus+ spines expressing GluA1, while empty arrowheads indicate spines without SA-PSDΔVenus or GluA1. Scale bar, 2 μm. **(E, F)** Comparison of spine volume **(E)** and GluA1 intensity **(F)** between SA-PSDΔVenus+ (SA+) and unlabeled (SA–) spines within the same tdTomato+ neurons after Gly-cLTP treatment. **(G)** Percentage of total spines with elevated GluA1 levels (defined as GluA1 intensity above the mean of unlabeled spines; GluA1+) and SA-PSDΔVenus+ (SA+) after Gly-cLTP treatment. **(H)** Percentage of SA-PSDΔVenus+ (SA+) and unlabeled (SA–) spines exhibiting elevated GluA1 levels after Gly-cLTP treatment. In E–H, circular markers indicate values corresponding to each neuron. *P < 0.05, ****P < 0.0001, paired Student's *t*-test, *n* = 18 (SA– and SA+) neurons. Bar graphs show mean ± SEM.

To map potentiated dendritic spines, we used a PSDΔ95–mVenus–HA fusion reporter under the translational control of *Arc* 5′ and 3′ UTRs (Figure 1A; see “Methods” section). The resulting construct, SA-PSDΔVenus, showed increased reporter protein expression at dendritic spines in primary hippocampal neuronal cultures following potentiation-inducing stimulations (Figures 1B–D). Compared to previously reported constructs (Gobbo et al., 2017), which exhibited some somatic signal *in vitro* but not *in vivo*, PSDΔVenus showed minimal somatic and dendritic shaft expression even in neuronal cultures, indicating improved spine specificity and enabling unambiguous detection of potentiated spines (Supplementary Figure 1A).

Synaptic potentiation was induced by mild depolarization using 10 mM KCl (Gobbo et al., 2017; Rienecker et al., 2020), rather than by the irreversible 55 mM KCl protocol. This treatment significantly increased the fraction of SA-PSDΔVenus+ dendritic spines compared with non-treated controls (Figures 1B, C; Supplementary Figure 1B). Conversely, blocking synaptic potentiation with the NMDA receptor (NMDAR) antagonist AP5 reduced the fraction of SA-PSDΔVenus+ dendritic spines (Figures 1B, C; Supplementary Figure 1B). In contrast, a control construct lacking the *Arc* 5′ and 3′ untranslated regions (UTRs; Figure 1A) expressed PSDΔVenus in nearly all dendritic spines regardless of treatment (Figure 1C; Supplementary Figure 1B).

Glycine-induced synaptic potentiation (Gly-cLTP; Figure 1B; see section Methods) increased spine volume and GluA1 levels in SA-PSDΔVenus+ spines compared to unlabeled spines (Figures 1D–F). The fraction of SA-PSDΔVenus+ spines was comparable to the fraction of dendritic spines with elevated GluA1 levels (above the mean GluA1 intensity of unlabeled spines; Figure 1G). Consistently, a significantly larger fraction of SA-PSDΔVenus-positive spines exhibited elevated GluA1 levels compared to unlabeled spines (Figure 1H). Together, these data indicate that SA-PSDΔVenus selectively labels dendritic spines consistent with potentiation in an activity- and NMDAR-dependent manner.

### Contextual fear conditioning increases SA-PSDΔVenus+ spines in the hippocampal CA1 neurons

**Figure 2:**
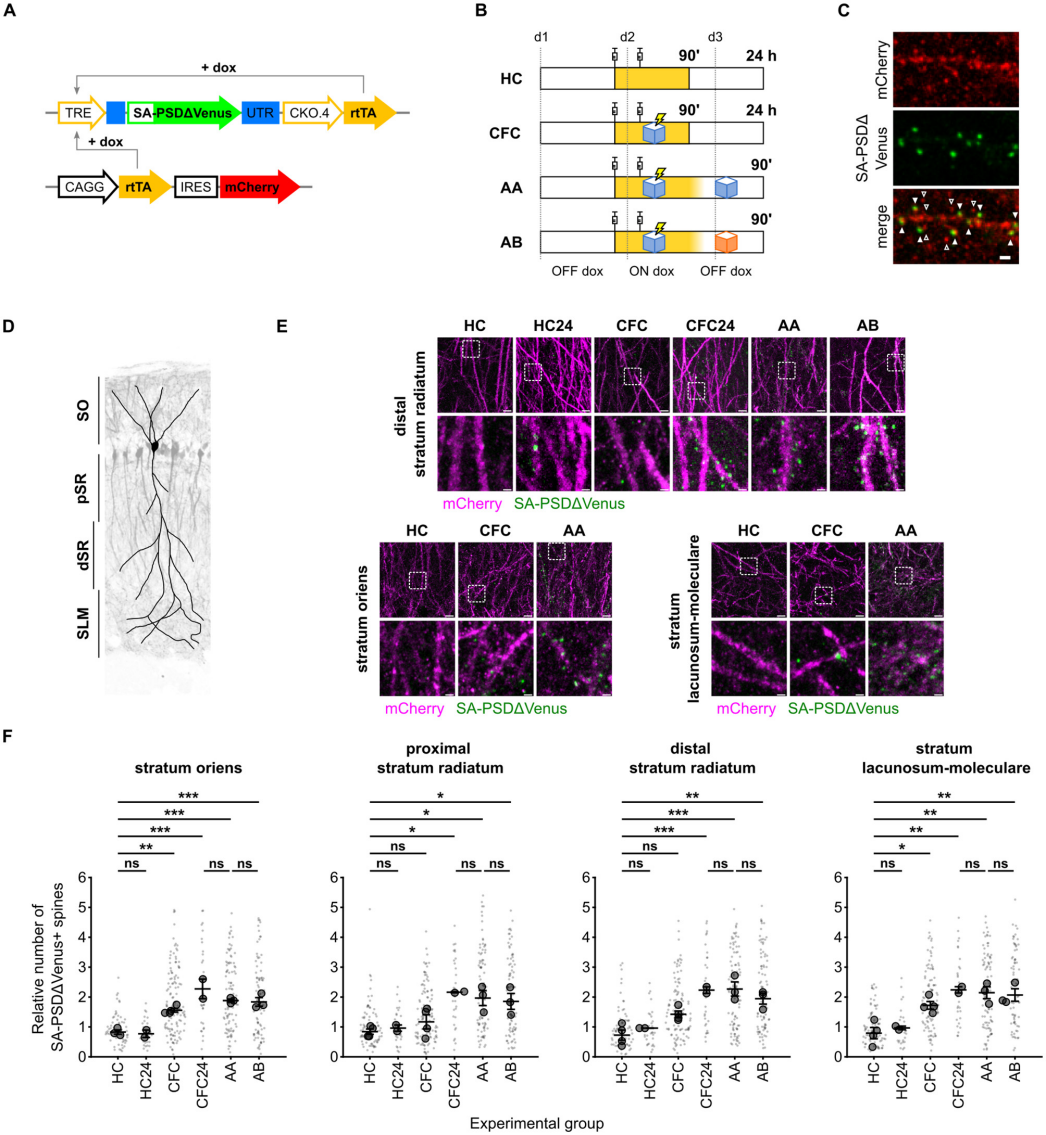
Contextual fear conditioning increases the number of SA-PSDΔVenus+ spines in the CA1 neurons. **(A)** Schematic of the constructs expressed in the hippocampal CA1 neurons. SA-PSDΔVenus is transcribed from the doxycycline-sensitive TRE promoter by the rtTA transcription factor (TET ON system). **(B)** Schematic of the four experimental groups. In yellow, the time window during which doxycycline is present after the injections is depicted. **(C)** Expression of neuron filler mCherry (red; anti-mCherry staining) and SA-PSDΔVenus (green; anti-HA staining) in the hippocampal CA1 neurons. Scale bar, 1 μm. Filled arrowheads indicate SA-PSDΔVenus+ spines, while empty arrowheads are spines without SA-PSDΔVenus. **(D)** Schematic of the regions considered in this study in the CA1 area, superimposed on the image of a mouse electroporated unilaterally in CA1 with CAG:TdTomato. SO, stratum oriens; pSR and dSR, proximal and distal portions of the stratum radiatum, respectively; SLM, stratum lacunosum-moleculare. **(E)** Representative images of the stratum oriens, distal stratum radiatum, and stratum lacunosum-moleculare from different experimental groups. Merged images of mCherry (magenta) and SA-PSDΔVenus (green) are shown for each group. Scale bar, 5 μm. The lower panel shows a magnified inset of the indicated region. Scale bar, 1 μm. **(F)** Relative number of SA-PSDΔVenus spines in different hippocampal CA1 layers for mice remained in the home cage (HC), subjected to contextual fear conditioning (CFC), and exposed to conditioned context A (AA) or to an unrelated context B (AB) 24 h after CFC. HC and CFC mice were perfused 90 min after the second dox injection, and HC24 and CFC24 animals were perfused 24 h after CFC. See **(B)** and text for details. Bar graphs show mean ± SEM. Smaller and larger circular markers indicate values corresponding to each dendrite and the average per animal, respectively. Statistical tests were performed with *N* as the number of animals. *P < 0.05, **P < 0.01, ***P < 0.001 and ns non-significant; one-way ANOVA, followed by Tukey's multiple comparison test; SO: *F*_(5, 11)_ = 20.69, *P* < 0.0001, pSR: *F*_(5, 12)_ = 6.900, *P* = 0.0030, dSR: *F*_(5, 12)_ = 14.53, *P* < 0.0001, SLM: *S*_(5, 12)_ = 12.12 P = 0.0002. *N* = 4 (HC, CFC), *N* = 3 (AA, AB), or *N* = 2 (HC24, CFC24) animals.

To examine the distribution of dendritic spines potentiated *in vivo* during memory formation, we expressed SA-PSDΔVenus in the hippocampal CA1 region of mice via triple-electrode *in utero* electroporation (Szczurkowska et al., 2016) and subjected them to contextual fear conditioning (CFC), a learning paradigm known to induce strong hippocampal neuronal activation and synaptic plasticity (Takeuchi et al., 2014; Josselyn and Tonegawa, 2020). Temporal control of SA-PSDΔVenus expression was achieved using a Tet-ON system (Figure 2A). Animals received two doxycycline injections to open a defined labeling window for learning-induced spine potentiation (Figure 2B). Mice subjected to CFC in context A were perfused either 90 min (CFC group) or 24 h (CFC24 group) after conditioning. Control mice remained in their home cages (HC) and underwent all experimental procedures, except fear conditioning (Figure 2B).

We observed SA-PSDΔVenus expression in the dendritic spines of mCherry+ hippocampal CA1 pyramidal neurons (Figure 2C). To assess layer-specific effects of contextual fear conditioning, we quantified the fraction of SA-PSDΔVenus+ spines on dendritic segments across the *stratum oriens* (SO), proximal and distal *stratum radiatum* (pSR and dSR), and *stratum lacunosum moleculare* (SLM; Figure 2D; see section Methods).

CFC mice exhibited a significant increase in the fraction of SA-PSDΔVenus+ spines compared to HC mice across all CA1 layers (Figures 2E, F; Supplementary Figure 2B). This increase was evident 90 min after CFC in the SO and SLM. In contrast, increases in the pSR and dSR were less pronounced at 90 min but became significant after 24 h. In HC mice, the fraction of SA-PSDΔVenus+ spines was similar at 90 min and 24 h (Figures 2E, F; Supplementary Figure 2B), indicating that the delayed increase observed in the SR of CFC mice is not due to prolonged doxycycline availability.

To determine whether context re-exposure further induces SA-PSDΔVenus expression, animals were re-exposed to the conditioned context A (AA) or to a different context (AB) 24 h after the fear-conditioning (Figure 2B). The fraction of SA-PSDΔVenus+ spines was comparable across the CFC24, AA, and AB groups in all CA1 layers (Figures 2E, F; Supplementary Figure 2B). Consistent with rapid doxycycline clearance (Lucchetti et al., 2019), this finding indicates that SA-PSDΔVenus labels spines potentiated during learning rather than during recall in AA and AB mice.

### ESARE-dTurquoise+ active neurons contain a higher fraction of SA-PSDΔVenus+potentiated spines following fear conditioning

**Figure 3:**
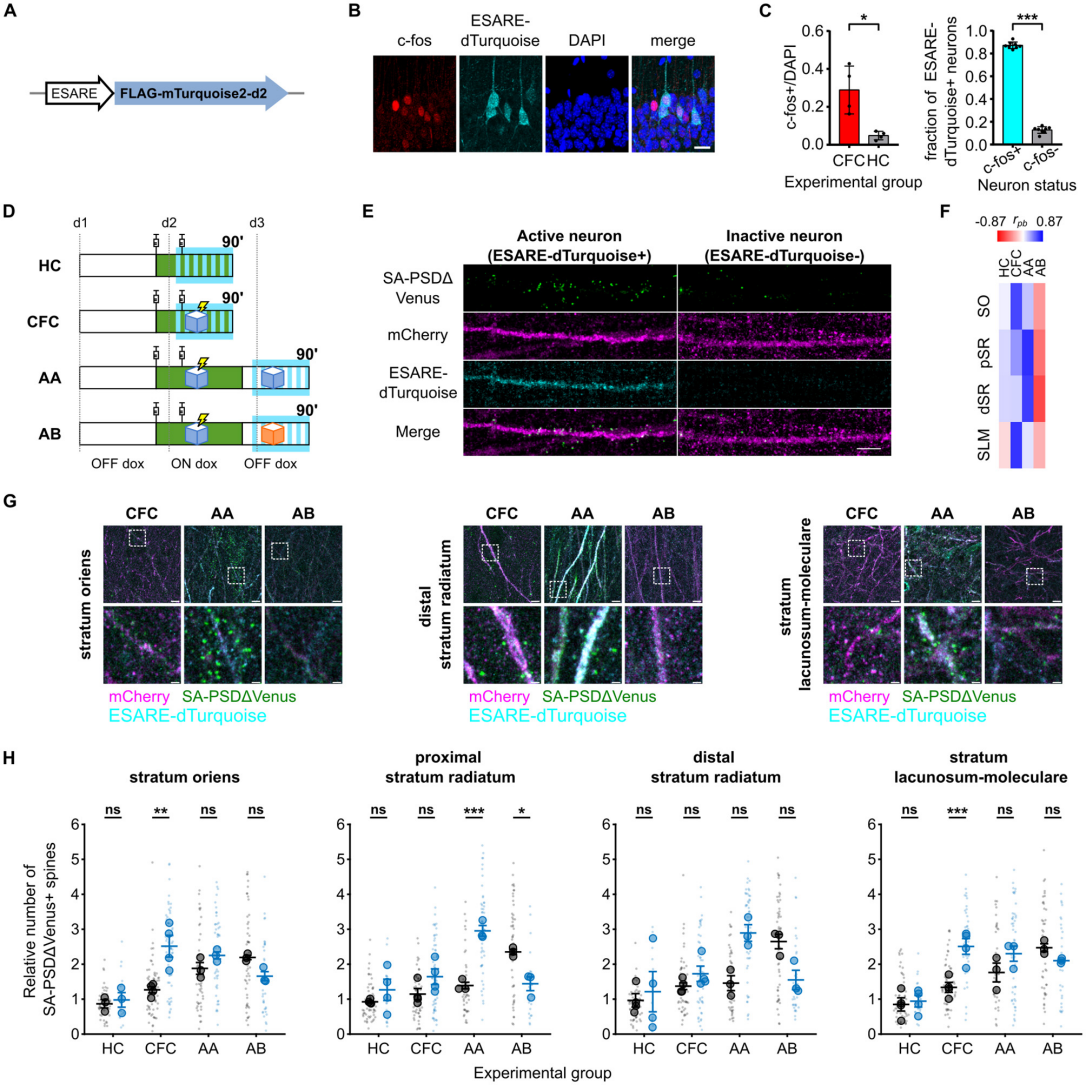
Dendritic spine potentiation overlaps with neuronal activation in the hippocampal CA1 following CFC. **(A)** Schematic of the construct expressed by neuronal activation. **(B)** ESARE-dTurquoise is expressed in c-fos+ neurons. Panels show FLAG staining (ESARE-dTurquoise), endogenous c-fos staining, and DAPI channels, scale bar 20 μm. Filled arrowheads are ESARE-dTurquoise+/c-fos+ cells, an empty arrowhead shows a faint ESARE-dTurquoise+ cell without c-fos staining, and an asterisk shows a c-fos+ cell without ESARE-dTurquoise expression. **(C)** Fraction of c-fos+ cells of all DAPI nuclei in the CFC and HC animals **(left)**. *P < 0.05 unpaired Student's *t*-test, Welch's correction for unequal variance (*t* = 3.757, *df* = 3.183), *N* = 4 animals. Fraction of ESARE-dTurquoise cells expressing c-fos in CFC animals **(right)**. ***P < 0.001 paired Student's *t*-test, *n* = 8 slices from *N* = 4 animals. **(D)** Schematic of the four experimental groups. Green depicts the time window during which doxycycline is present from the injections, and SA-PSDΔVenus is expressed at potentiated spines. Cyan depicts the time window of ESARE-dTurquoise expression on neurons activated. **(E)** Differential distribution of SA-PSDΔVenus+ spines in stratum radiatum dendrites of ESARE-dTurquoise+ (active) and ESARE-dTurquoise- (inactive) neurons in the AA group. SA-PSDΔVenus (green), mCherry (magenta), ESARE-dTurquoise (cyan), and merge are shown for each group. Scale bar, 5 μm. **(F)** Point biserial correlation (rpb) between the number of SA-PSDΔVenus+ spines and neuronal activation status (ESARE-dTurquoise+) in different layers for the four groups (see also Supplementary Table 1). **(G)** Representative images of stratum oriens, stratum radiatum, and stratum lacunosum moleculare for the CFC, AA, and AB groups. Merged images of mCherry (magenta), ESARE-dTurquoise (cyan), SA-PSDΔVenus (green) are shown for each group. Scale bar, 5 μm. The lower panel shows a magnified inset of the indicated region. Scale bar, 1 μm. **(H)** Relative number of SA-PSDΔVenus+ spines in ESARE-dTurquoise- (black) or ESARE-dTurquoise+ (blue) neurons in different layers as in Figure 2. Smaller and larger circular markers indicate values corresponding to each dendrite and the average per animal, respectively. Statistical tests were performed with *N* as the number of animals. *P < 0.05, **P < 0.01, ***P < 0.001 and ns non-significant, two-way ANOVA, followed by Bonferroni's multiple comparison test; SO: interaction: *F*_(3, 9)_ = 8.217, *P* = 0.0060, pSR: interaction *F*_(3, 10)_ = 17.09, *P* = 0.0003, dSR: interaction *F*_(3, 10)_ = 4.568, *P* = 0.0291 and SLM: interaction *F*_(3, 10)_ = 9.370, *P* = 0.0030. *N* = 4 (HC, CFC) or *N* = 3 (AA, AB) animals. Bar graphs show mean ± SEM (**C, E**).

To identify activated neurons, we co-electroporated a third construct (ESARE-dTurquoise) expressing a fusion protein comprising an N-terminal FLAG tag, mTurquoise2, and a C-terminal destabilization tag under the control of the enhanced synaptic activity-responsive element (ESARE) promoter (Figure 3A). ESARE is an engineered version of the activity-dependent Arc promoter (Kawashima et al., 2013), and the C-terminal destabilization tag shortens the protein half-life, aligning reporter expression with endogenous immediate-early gene (IEG) dynamics (Li et al., 1998; Wang et al., 2006; Eguchi and Yamaguchi, 2009; Attardo et al., 2018). Consistent with this design, ESARE-dTurquoise showed a strong overlap with the endogenous IEG c-fos (Figure 3B). More than 80% of c-fos+ hippocampal CA1 neurons expressed ESARE-dTurquoise, indicating that it reliably reports neuronal activation (Figure 3C). Notably, because a small fraction of c-fos+ neurons lacked ESARE-dTurquoise, some ESARE-dTurquoise- neurons may represent active but non-transfected cells.

Next, we analyzed the distribution of SA-PSDΔVenus+ spines in hippocampal CA1 neurons that had been active (mCherry+/ESARE-dTurquoise+) or inactive (mCherry+/ESARE-dTurquoise-) 90 min after CFC (Figures 3D, E). In the SO and SLM layers, we observed a higher fraction of SA-PSDΔVenus+ spines in ESARE-dTurquoise+ neurons compared to ESARE-dTurquoise- neurons, indicating a correlation between neuronal activation and synaptic potentiation (Figures 3F, G, H; Supplementary Figure 3A). In HC mice, the fraction of SA-PSDΔVenus+ spines was comparable between active and inactive neurons, and the number of ESARE-dTurquoise+ neurons was low (Figures 3G, H; Supplementary Figure 3A).

We next re-exposed a group of CFC mice to the conditioned context A 24 h after CFC (AA group). In this condition, ESARE-dTurquoise reports neuronal activation induced by context re-exposure, whereas SA-PSDΔVenus labels dendritic spines potentiated during CFC (Figure 3D). Context re-exposure does not induce SA-PSDΔVenus expression (Figures 2E, F; Supplementary Figure 2B), consistent with doxycycline being cleared from the brain within 24 h after administration (Lucchetti et al., 2019). In the pSR and dSR layers, the fraction of SA-PSDΔVenus+ spines was higher in ESARE-dTurquoise+ neurons compared to ESARE-dTurquoise- neurons (Figures 3G, H; Supplementary Figure 3A). In contrast, CFC mice re-exposed to a different context B (AB group) showed the opposite pattern (Figures 3D, G, H; Supplementary Figure 3A). In the AB group, the fraction of SA-PSDΔVenus+ spines was lower in the ESARE-dTurquoise+ neurons compared to the ESARE-dTurquoise- neurons in the pSR and dSR layers (Figures 3G, H; Supplementary Figure 3A).

In summary, neuronal activation correlated positively with synaptic potentiation in CFC and AA mice but negatively in AB mice (Figure 3F; Supplementary Table 2). In CFC mice, this correlation was strongest in the SO and SLM, whereas, in AA mice, it was most pronounced in the pSR and dSR.

## Discussion

Experimental advances in neuroscience have enabled the identification of cellular engrams—neuronal ensembles whose activation is necessary and sufficient for memory retrieval (Andersen et al., 2007; Josselyn and Tonegawa, 2020). However, most engram-labeling strategies rely on immediate-early gene (IEG) reporters that label entire neurons, providing limited insight into which specific synapses within those neurons are modified during learning (Andersen et al., 2007; Takeuchi et al., 2014). As a result, the relationship between cellular engrams and synapse-specific plasticity remains incompletely understood (Choi et al., 2018; Kim et al., 2025). Here, we combined activity-dependent reporters for neuronal activation (ESARE-dTurquoise) and translation-dependent synaptic potentiation (SA-PSDΔVenus) to directly examine how learning-induced synaptic plasticity is distributed across activated and non-activated neurons during contextual fear conditioning (CFC).

In primary hippocampal neurons, SA-PSDΔVenus selectively labeled dendritic spines consistent with NMDAR-dependent synaptic potentiation (Figure 1C). Labeled spines exhibited increased volume and elevated GluA1 levels (Figures 1D–H), consistent with established structural and molecular correlates of long-term potentiation (Matsuzaki et al., 2004). These effects are unlikely to reflect overexpression artifacts, as similar outcomes were observed with earlier SynActive constructs lacking PSD95 fusion domains (Gobbo et al., 2017), and the truncated PSD95 used in this study reduces synaptic perturbation while preserving spine targeting (Arnold and Clapham, 1999; Hayashi-Takagi et al., 2015).

Extending this approach *in vivo*, we found that CFC induced a significant increase in SA-PSDΔVenus+ spines in hippocampal CA1 neurons compared with home-cage controls (Figures 2E, F). This increase displayed layer- and time-dependent dynamics, with rapid potentiation in the *stratum oriens* (SO) and *stratum lacunosum moleculare* (SLM) and a delayed increase in the *stratum radiatum* (SR). These observations are consistent with prior evidence that memory consolidation involves post-learning reactivation of CA1 circuits during sleep and quiet wakefulness, particularly along the CA3–CA1 pathway (Sadowski et al., 2016; Wu et al., 2017; Yang et al., 2024). We therefore speculate that delayed synaptic potentiation in the SR reflects consolidation-related plasticity, consistent with reports of stepwise synaptic plasticity and delayed Arc expression contributing to memory persistence (Nakayama et al., 2015; Goto et al., 2021; Das et al., 2023).

By jointly labeling activated neurons (ESARE-dTurquoise+) and potentiated synapses, we demonstrate that neurons activated during learning contain a higher fraction of SA-PSDΔVenus+ spines than inactive neurons (Figures 3G, H). This finding directly links learning-induced neuronal activation to the spatial distribution of synaptic plasticity within individual neurons. Previous studies have reported increased spine density, enhanced synaptic strength, and LTP occlusion in neurons activated during fear conditioning (Takahashi et al., 2012; Ryan et al., 2015; Choi et al., 2018), whereas our approach enables direct visualization of learning-induced potentiated synapses at single-spine resolution *in vivo*.

Reactivation of learning-activated neurons during recall has been widely documented using IEG labeling, electrophysiology, and calcium imaging, although the overlap between encoding and recall ensembles is often incomplete (Ramirez et al., 2014; Cai et al., 2016; Ólafsdóttir et al., 2018; Ghandour et al., 2019). Our findings suggest that this reactivation is biased toward neurons containing a higher fraction of learning-induced potentiated spines, particularly within the SR (Figures 3G, H). This interpretation is consistent with studies showing that disruption of synaptic plasticity in learning-activated neurons impairs neuronal reactivation and memory recall (Ryan et al., 2015; Kitamura et al., 2017; Abdou et al., 2018).

It is important to distinguish reporters of synaptic activity from those labeling translation-dependent long-term potentiation, a feature currently unique to SynActive-based approaches (Hayashi-Takagi et al., 2015; Gobbo et al., 2017; Gobbo and Cattaneo, 2020). Regardless this distinction, multiple studies have linked synaptic activity and neuronal recruitment during learning using complementary strategies, including AMPAR pulse–chase labeling, dual-eGRASP, and photoconvertible synapse reporters (Choi et al., 2018; Perez-Alvarez et al., 2020; Kim et al., 2025). In agreement with these findings, we observe a positive correlation between neuronal activation and the localization of potentiated synapses in CFC mice. Recent studies have demonstrated that branch-specific dendritic plasticity, in addition to neuronal ensemble overlap, is crucial for memory linking (Frank et al., 2018; Sehgal et al., 2025). Integrating SynActive into such approaches could further increase the resolution of memory allocation from neurons and dendritic branches to individual spines or help identify “dendritic engrams” (Kastellakis et al., 2023) by measuring the number or density of potentiated spines on individual branches. Future research combining SynActive reporters with viral delivery systems and longitudinal imaging could enable whole-brain, multi-area mapping of synaptic potentiation *in vivo*.

Despite the potential of the SynActive strategy, several limitations should be acknowledged. First, we did not assess the persistence or turnover of SA-PSDΔVenus labeling, leaving open the question of whether tagged spines are maintained long-term or replaced by new potentiation events. Second, although destabilized reporters are designed to reflect transient activation (Li et al., 1998; Eguchi and Yamaguchi, 2009), their precise degradation kinetics and diffusion properties in distal dendrites require further characterization. Third, ESARE-dTurquoise expression may be influenced by novelty or stress associated with context exposure, potentially contributing to neuronal activation independent of learning or recall. Fourth, how the number or spatial distribution of potentiated spines relates to memory strength (e.g., freezing behavior) remains to be determined.

In summary, this study provides synaptic-resolution evidence linking neuronal activation across different phases of associative memory to the localization of learning-induced potentiated spines within individual neurons. These findings may inform computational models of dendritic integration and synaptic plasticity (Kastellakis et al., 2016) and extend to studies of memory mechanisms in both physiological and pathological contexts.

## Data Availability

The original contributions presented in the study are included in the article/Supplementary material, further inquiries can be directed to the corresponding author.
