# Supplementary material for: Distinct spatial distribution of potentiated dendritic spines in encoding- and recall-activated hippocampal neurons
Source: Front Mol Neurosci. 2026 Jan 20;18:1751677. doi: 10.3389/fnmol.2025.1751677 (PMC12864489; doi:10.3389/fnmol.2025.1751677)
Supplement: Supplementary file 1 [file Data_Sheet_1.docx]

**Distinct spatial distribution of potentiated dendritic spines in encoding- and recall-activated hippocampal neurons**

Francesco Gobbo, Ajesh Jacob, Bruno Pinto, Marco Mainardi, Laura Cancedda and Antonino Cattaneo

## SUPPLEMENTAL INFORMATION

| \| 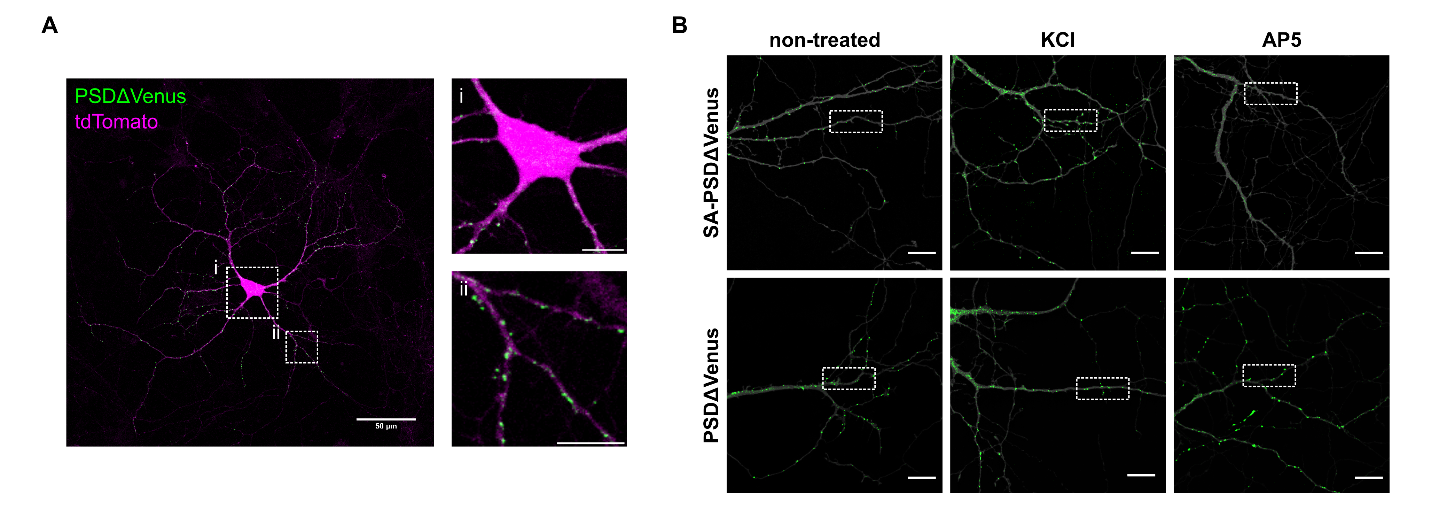 \| \| --- \| |
| --- | --- |

### Supplementary Figure 1. PSDΔVenus labels dendritic spines in primary hippocampal neurons.

(A) Expression of PSDΔVenus in primary hippocampal neurons (green) along with tdTomato (magenta). The signal is confined to dendritic spines and absent from axons and soma. Scale bar, 50 μm; inset: 10 μm.

(B) Lower-magnification views of the images shown in Figure 1C. Primary mouse hippocampal neurons expressing tdTomato (greyscale) and SA-PSDΔVenus or PSDΔVenus (green) after 24 h D-AP5 (AP5), 90 min KCl (KCl) or non-treated (nt). Scale bar, 10 μm.

| \| 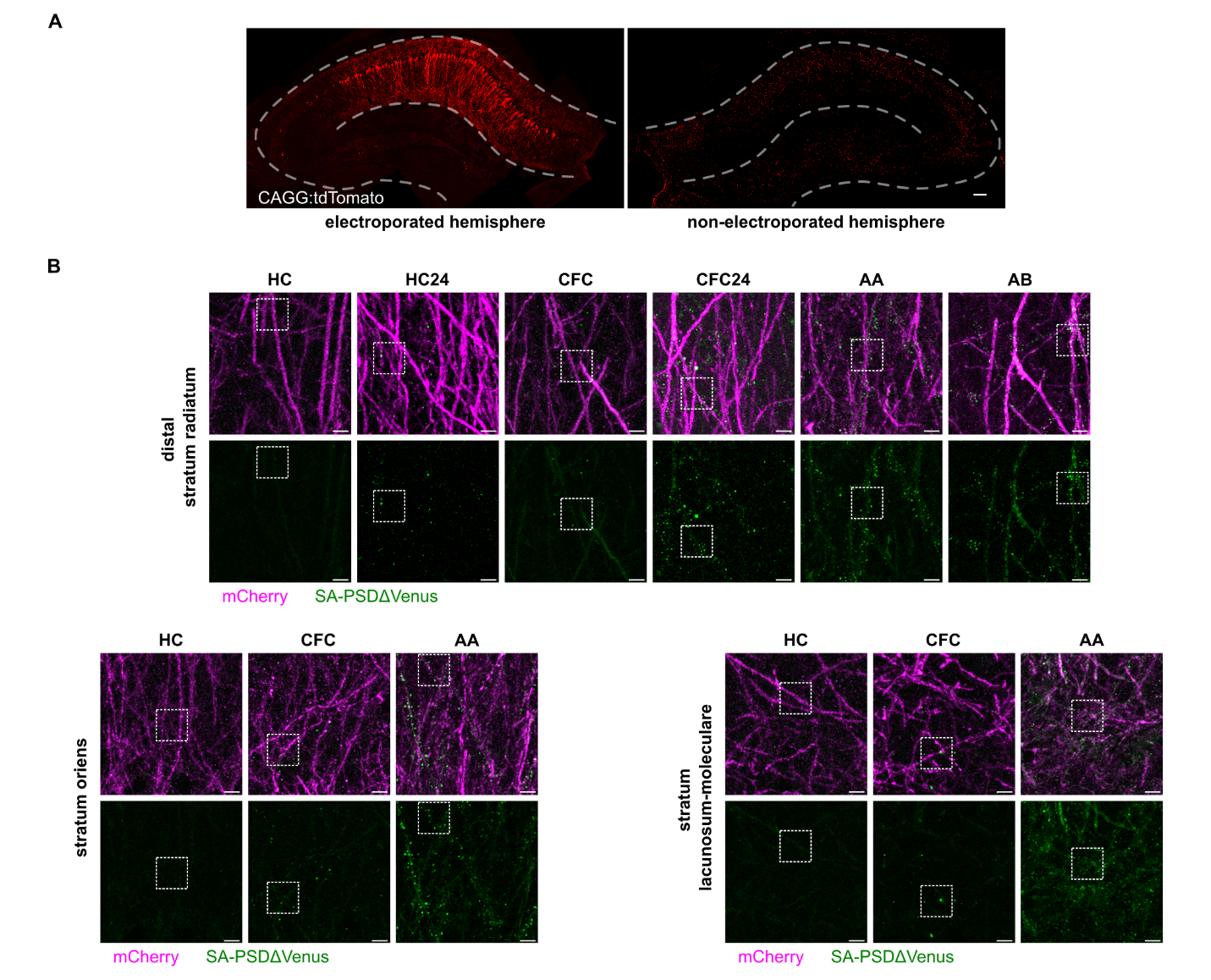 \| \| --- \| |
| --- | --- |

### Supplementary Figure 2. Contextual fear conditioning increases the number of SA-PSDΔVenus+ spines in the CA1 neurons.

(A) Low-magnification image showing in utero electroporation efficiency. Animals were unilaterally electroporated in CA1 with CAGG:tdTomato. Cell bodies and dendrites are visible on the electroporated side, whereas axonal projections are observed in the non-electroporated hemisphere. Scale bar, 100 μm.

(B) Same images as in Figure 2E, additionally showing the individual SA-PSDΔVenus channel. Representative images of the *stratum oriens, distal stratum radiatum* and *stratum lacunosum- moleculare* from different experimental groups. Merged images of mCherry (magenta) and SA-PSDΔVenus (green) are shown for each group. Scale bar, 5 μm.

| 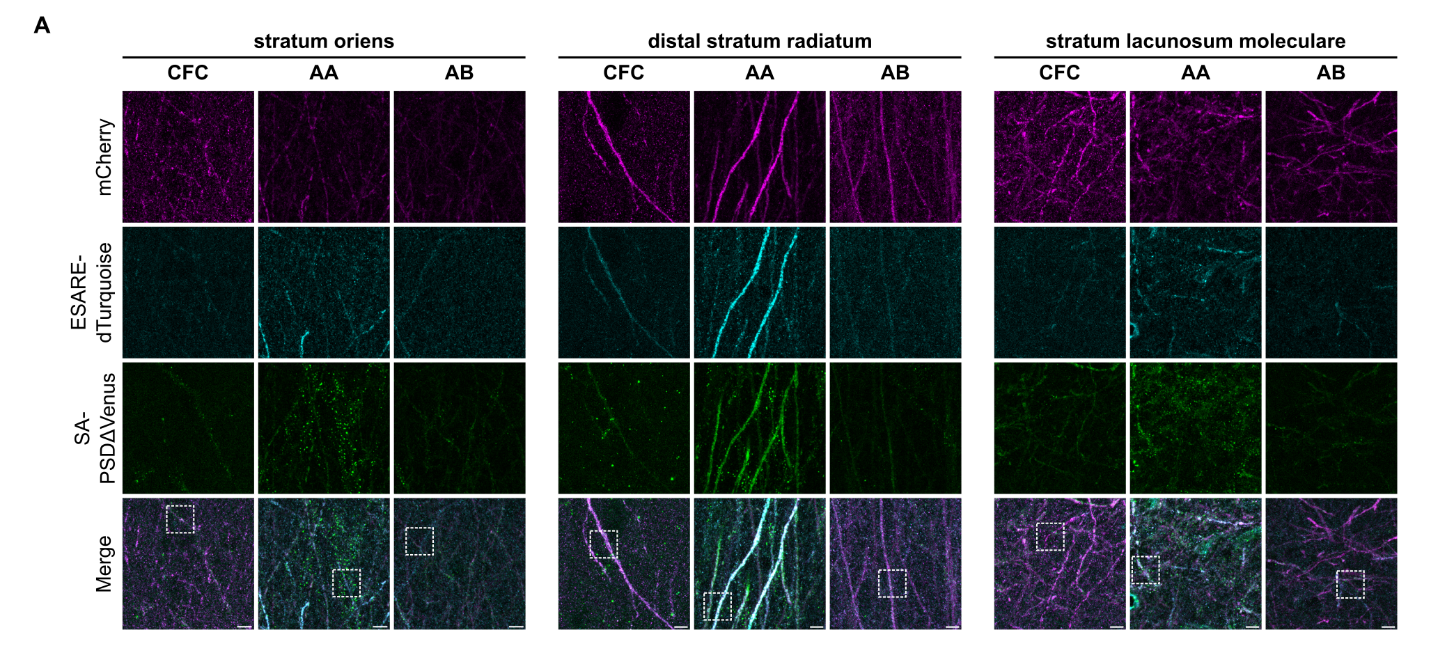 |  |
| --- | --- |

### Supplementary Figure 3. Dendritic spine potentiation overlaps with neuronal activation in the hippocampal CA1 following CFC.

(A) Same images as in Figure 3G, additionally showing the individual channels. Representative images of *stratum oriens, stratum radiatum* and *stratum lacunosum moleculare* for the CFC, AA and AB groups. Merged images of mCherry (magenta), ESARE-dTurquoise (cyan), SA-PSDΔVenus (green) are shown for each group. Scale bar, 5 μm.

|  |
| --- |

### Supplementary Table 1. Sequences of PSDΔ, 5’ and 3’ Arc untranslated regions (UTRs).

| **Insert** | **Sequence** |
| --- | --- |
| PSDΔ | ATGGACTGTCTCTGTATAGTGACAACCAAGAAATACCGCTACCAAGATGAAGACACGCCCCCTCTGGAACACAGCCCGGCCCACCTCCCCAACCAGGCCAATTCTCCCCCTGTGATTGTCAACACGGACACCCTAGAAGCCCCAGGATATGAGTTGCAGGTGAATGGAACAGAGGGGGAGATGGAGTATGAGCGGATCGTGATCCATCGGGGCTCCACCGGCCTGGGCTTCAACATCGTGGGCGGCGAGGATGGTGAAGGCATCTTCATCTCCTTCATCCTTGCTGGGGGTCCAGCCGACCTCAGTGGGGAGCTACGGAAGGGGGACCAGATCCTGTCGGTCAATGGTGTTGACCTCCGCAATGCCAGTCACGAACAGGCTGCCATTGCCCTGAAGAATGCGGGTCAGACGGTCACGATCATCGCTCAGTATAAACCAGAAGAGTATAGTCGATTCGAGGCCAAGATCCATGATCTTCGGGAACAGCTCATGAATAGTAGCCTAGGCTCAGGGACTGCATCCTTGCGAAGCAACCCCAAGAGGGGCTTCTACATTAGGGCCCTGTTTGATTACGACAAGACCAAGGACTGCGGTTTCTTGAGCCAGGCCCTGAGCTTCCGCTTCGGGGATGTGCTTCATGTCATTGACGCTGGTGACGAAGAGTGGTGGCAAGCACGGCGGGTCCACTCCGACAGTGAGACCGACGACATTGGCTTCATTCCCAGCAAACGGCGGGTCGAGCGACGAGAGTGGTCAAGGTTAAAGGCCAAGGACTGGGGCTCCAGCTCTGGATCACAGGGTCGAGAAGACTCGGTTCTGAGCTATGAGACGGTGACCCAGATGGAAGTGCACTATGCTCGTCCCATCATCATCCTTGGACCCACCAAAGACCGTGCCAACGATGATCTTCTCTCCGAGTTCCCCGACAAGTTTGGATCCTGTGTCCCTCATACGACACGTCCTAAGCGGGAATATGAGATAGACGGCCGGGATTACCACTTTGTCTCCTCCCGGGAGAAAATGGAGAAGGACATCCAGGCACACAAGTTCATTGAGGCTGGCCAGTACAACAGCCACCTCTATGGGACCAGCGTCCAGTCTGTGCGAGAGGTAGCAGAGCAGGGGAAGCACTGCATCCTCGATGTCTCGGCCAATGCCGTGCGGCGGCTGCAGGCGGCCCACCTGCACCCCATCGCCATCTTCATCCGTCCCCGCTCCCTGGAGAATGTGCTAGAGATCAATAAGCGGATCACAGAGGAGCAAGCCCGGAAAGCCTTCGACAGAGCCACGAAGCTGGAGCAGGAGTTCACAGAGTGCTTCTCAGCCATCGTAGAGGGCGACAGCTTTGAAGAGATCTATCACAAAGTGAAACGTGTCATTGAAGACCTCTCAGGCCCCTACATCTGGGTCCCAGCCCGAGAGAGACTC |
| 5’ *Arc* UTR | AGTGCTCTGGCGAGTAGTCCTCCCTCAGCCGCAGTCTCTGGGCCTCTTCAGCTTGAGCGGCGGCGAGCCTGCCACACTCGCTAAGCTCCTCCGGCACCGCGCACTTGCCACTGCCACTGCCGCTTCGCGCCCGCTGCAGCCGCCGGCTCTGAATCCTTCTGGCTTCCGCCTCAGAGGAGTTCTTAGCCTGTCCCGAACCGTAACCCCGGCGAGCAGACGGAGCTGGACCA |
| 3’ *Arc* UTR | TCCCCAGCCTGCCTGCCACACCCAGTCTGTGGCTTTTGTCAACTAGGACTTGATTGAGCTGGGGCTGACACCCAAGGGGATGCCCTGTCCAGCCAGACACCTTCTCACCCACTGGCCTGACTCACAACTGCCACACAACCATGATTCATGGACATCAAGAAGCCCCTCTCCCATAGGGCTCCCACCTGCCACCTACCCCTCACCTGTCTGCCCTAGTCCTGGCCCTGTCTCCAGTGGCCTCACCCTCTACACTCTCAGACCATCACAGAACACCTTTGGCTTCCTCATTCTGCATCAGTGTCCAGGGCCCTTTGGGTAGTCAAGAAATCAAGTGTCTGAAAGGCAATGAAAAGTAGGCACCAAACCCAAGGGGCATCCCAGGGCAGATGCTAAAGCAGAATCAGAGATGGCCGAAGGAACCTCTACTTCCGGGGATGCAGCCCGCTCCTACAGACACAGCAGATCCAGCTGGTGCCCTACCTGCCTCCCAGAGCAACTGGCCAGTCTTGGGCAGCATAGCTCCCCTCTCAGGGTGAGCTGAAGCAGCAGACCTGACGCGCTGGCGCCTCCTGGCCCCCAGCAGTGATTCATACCAGTGAAGAAAAGCAGACTTCGGCTCCATGACTCAGCCATGCCAGGCGGAGGGTCCCAGAGGGGCTGAGTCCTCAGCCCCAGCTGAGGCAGCAGCTGGAGTCTTCAGAGCCAGGTGAATGACACCAGGTCTCAAGCTGCTGAGAAGTCTTTCCGGCCATGTCTGGAAGGGGTACCACCCCAGCACCAGCACCGTCCCCTCCTCTCTTGAAGCTGCCTGCACAGAGGTTCCAAGACACTTTCAAGGCAGAGAAAATAGGATTACAAAGAGGAGGTGCCTGGCAGAGGGCAGCACCCAGCTCAGCCTCAGAGCTGAAGGTGAAGACAAGCCAGCGTGAAACCCCGGGTCTGCCACGAATGCCCGCTCCGCTGGCCACTCACCAGCTGCCTGCCACAAGCCACTGCAGCTTGAGCAGGGTCTGTGCCCTCTCAGCACAGAGCCCAGTTCGCTGCGTGGCCTTTGGCCCCCGCCAGAACCTTGCAGGAGCCTTAAGGTTCGGGCCCTAGCCCAGCCTGACCTTACCTGCTGTGCCCTGCCTGCTGGTCAAGTCCAGTCCCAGGAGACCCCATGCCTTGGCTCCTAGGCTGTTCCAGGCACTTCCCTGACCTGCCGGGTGATTGCCCAGCTGGAACCTCATCCACACCCCAGCACCAACCACCTCGTGTTGGTAACTGCTCGTGTCTGTAGTCTGAGTAGGCCATGTTGAGGTTCCTCCATCTGCCTGGTCCATTGGTGTTCTGAGACCAGTTCCACTGCTGTTCTGACAGATCCCCCACCCTGTGCCCCTGCCAGCCCCCACAGGTTTATTTTTGCACATAAACCATGACCCATACTAATTTGGCTAGCTCTGGGGACTAGGGAGACCCTGGAGATCTCAAGAGTGTGGCTATCCCCTATTTTCACCAAGCCTTCAATATCCAGCCAGGCCATCTGCCCACACCATCTTACCTCAAAGACAGACATATATATATATATACATATATATGATTTTGTTAATAAAACTATGAAATTTAAA |

### Supplementary Table 2. Point biserial correlation (*rpb*) between the number of SA-PSDΔVenus+ spines and neuronal activation status (ESARE-dTurquoise+) in different layers for the four experimental groups.

| **Group** | **Layer** | **Point Biserial correlation** | **95% Confidence Interval** |
| --- | --- | --- | --- |
| HC | SO | 0.1267 | -0.1271 to 0.3649 |
|  | pSR | 0.1852 | -0.03897 to 0.3916 |
|  | dSR | 0.1913 | -0.04941 to 0.411 |
|  | SLM | -0.02052 | -0.2479 to 0.209 |
| CFC | SO | 0.5331 | 0.3968 to 0.6464 |
|  | pSR | 0.3412 | 0.1686 to 0.4934 |
|  | dSR | 0.1943 | 0.008178 to 0.3674 |
|  | SLM | 0.5804 | 0.4291 to 0.7001 |
| AA | SO | 0.2323 | 0.03963 to 0.4083 |
|  | pSR | 0.5917 | 0.4412 to 0.7099 |
|  | dSR | 0.5565 | 0.406 to 0.6776 |
|  | SLM | 0.1554 | -0.05222 to 0.3501 |
| AB | SO | -0.2257 | -0.4166 to -0.01585 |
|  | pSR | -0.3424 | -0.5149 to -0.1432 |
|  | dSR | -0.382 | -0.5477 to -0.1876 |
|  | SLM | -0.1463 | -0.3586 to 0.08029 |

### Supplementary Methods

#### Primary hippocampal cultures

Primary hippocampal neurons were prepared as previously described (Gobbo et al., 2017). Hippocampi from P0 B6126 mice were microdissected under sterile conditions and triturated in cold calcium-free Hank’s balanced salt solution (HBSS) (Sigma-Aldrich H6648) 100 U/ml penicillin/0.1 mg/ml streptomycin (Thermo Fisher 15070063) and digested in 0.1% trypsin (Thermo Fisher 15090046), followed by inactivation in 10% FBS (Thermo Fisher 10500064) in DMEM (Invitrogen 11880028) 100 U/ml DNase (Sigma-Aldrich D5025). Dissociated neurons were pelleted by centrifugation at 1000 rpm for 5 min, followed by resuspension in Neurobasal-A (Thermo Fisher 10888022) supplemented with 4.5 g/L D-glucose (Sigma Aldrich G7021), 10% FBS (Thermo Fisher 10500064), 2% B27 (Thermo fisher 17504044), 1% Glutamax (Thermo Fisher 35050061), 1 mM pyruvate (Sigma Aldrich S8636) and 12.5 μM glutamate (Sigma Aldrich G5889). Neurons were seeded on 24-well plates containing 13 mm glass coverslips coated with Poly-D-Lysine (PDL,) or on PDL-coated, plasma-treated Willco dishes (Willco Wells GWST-3522). The day after seeding, the medium was changed to Neurobasal-A supplemented with 2% B27, 1% Glutamax and 10 μg/ml gentamicin (neuronal growth medium). On the day in vitro (div) 2, 2.5 μM AraC was added to reduce glia proliferation. The culture medium was refreshed every 2 days.

#### Calcium phosphate transfection

Neurons were transfected with the calcium phosphate method the day before the experiment: 10μg DNA is dissolved in 100μl 250 mM CaCl2 (Sigma Aldrich C3306), then 100μl of 2xHBS (280 mM NaCl (Sigma Aldrich S9888), 50 mM HEPES (Sigma Aldrich H4034), 1.4 mM Na2HPO4 (Sigma Aldrich S7907) pH 7.1) are added dropwise while vortexing. After 20 minutes the resulting suspension is added to the well. After 90 minutes, the medium is removed, cultures washed with 1mM MgCl_2_ 2mM CaCl_2_ HBSS and 1:1 fresh:conditioned culture medium is added to the neurons.

#### Lipofectamine transfection

According to the manufacturer's instructions the protocol followed for a well in a 24-well plate is as follows. The conditioned medium was collected from the well, and neurons were washed with warm HBSS containing 2 mM CaCl2 and 1 mM MgCl2. Neurons were then placed back in the incubator after adding 400 µl of fresh neuronal growth medium. pAAV-TRE3-SA-PSDΔVenus (1 µg)/pAAV-hSyn-rtTA-P2A-tdTomato (1µg) plasmid mix was then diluted in Opti-MEM to 50 µl and mixed with Lipofectamine 2000 (3 µl) diluted in 50 µl Opti-MEM. After incubation at room temperature for 5 minutes, the 100 µl transfection mix was added to the neurons and incubated for 2.5 hours at 37°C. Finally, the transfection mix-containing medium was replaced with the conditioned medium (350 µl) and fresh neuronal growth medium (150 µl).

#### Glycine-mediated chemical LTP (Gly-cLTP)

Gly-cLTP was induced in cultured hippocampal neurons between div14 -16 as previously described (Lu et al., 2001). Conditioned medium was collected, and the neurons were initially incubated in extracellular solution (ECS, pH 7.4, containing 140 mM NaCl, 1.3 mM CaCl2, 5 mM KCl, 25 mM HEPES, 33 mM D-glucose, 0.5 µM tetrodotoxin (TTX, Tocris 1078), 1 µM strychnine (Sigma-Aldrich) and 50 µM picrotoxin (PTX, Tocris 1128) at 37°C for 30 min. The solution was then replaced with ECS containing 200 µM Glycine (Sigma-Aldrich) to induce LTP. After 3 min, the solution was switched back to normal ECS and neurons were incubated at 37°C for another 30 min. Finally, ECS was removed, and the conditioned medium aspirated at the beginning of the experiment was added back. The neurons remained in the 37°C incubator until fixation at 90 minutes post Gly-cLTP.

#### Immunolabeling of neuronal cultures

Neuronal cultures fixed with 4 % PFA were permeabilized in 0.1% Triton X-100, 2.5% bovine serum albumin (BSA) PBS for 7 min, followed by five washes with PBS and blocking in 5% BSA PBS for 1 h. Neurons were then incubated with rabbit monoclonal GluA1 antibody (Cell signaling 13185, 1:500) in 2.5% BSA PBS for 2-3 h at room temperature. After washing thrice with PBS, neurons were incubated with anti-rabbit-647 (Thermo Fisher A-31573) in 2.5% BSA PBS for 1 h at room temperature. Neurons were washed thrice in PBS and once in the water, and the glass coverslips with fixed neurons were then mounted using Vectashield antifade (Vector Laboratories) mounting media.

#### Immunolabeling of Brain slices

After perfusion, brains were post-fixed overnight in 4% formaldehyde in PBS, then cryoprotected in 30% sucrose PBS. Sixty μm-thick coronal sections were cut with a cryostat. After washing the slices in PBS, slices were blocked for 1h in PBS 0.3% Triton X-100 (Sigma-Aldrich T8787), 10% filtered FBS (Thermo Fisher 10500064), then incubated with primary antibodies goat anti-HA (Santa Cruz, sc-805-g) 1:100 / rabbit anti mCherry (Abcam ab167453) 1:200 / mouse anti-FLAG (Sigma-Aldrich F3165) in PBS 0.3% Triton X-100, 10% FBS overnight at 4°C shaking. After incubation, slices were washed three times for 10 minutes each with PBS 10% FBS 0.1 % Triton x-100, then incubated with 1:200 donkey anti-goat Alexa Fluor488 (Thermo Fisher A-11055)/ 1:200 donkey anti-Rabbit Alexa Fluor555 (Thermo Fisher A-31572)/ 1:200 donkey anti-mouse Alexa Fluor647 (Thermo Fisher A-31571) in PBS 0.1% Triton X-100 10% FBS for three hours at room temperature upon shaking. Slices were then incubated for ten minutes in PBS Triton X-100 0.1% with 10 μg/ml DAPI (Sigma-Aldrich D9542), then washed three times in PBS for 10 minutes, and once in deionized water before mounting them in VECTASHIELD Antifade Mounting Medium (Vector Laboratories H-1000).

To compare the overlap between ESARE-dTurquoise and endogenous c-fos expression, slices from HC or CFC groups were stained for c-fos/FLAG immunoreactivity as follows: 1 h blocking in PBS 0.3% Triton X-100 (Sigma-Aldrich T8787), 10% normal goat serum (NGS) (Sigma Aldrich NS02L), then incubated with primary antibodies mouse anti-FLAG (Sigma-Aldrich F3165) / rabbit anti-c-fos (Santa Cruz, sc-52) 1:100 in PBS 0.1% Triton X-100, 10% NGS overnight at 4°C shaking. After three washes for 10 minutes each with PBS, slices were incubated with 1:200 donkey anti-mouse Alexa Fluor488 (Thermo Fisher A-21202) / 1:200 donkey anti-rabbit Alexa Fluor647 (Thermo Fisher A-31573) in PBS 0.1% Triton X-100 for three hours at room temperature upon shaking. Slices were then incubated for ten minutes in PBS Triton X-100 0.1% with 10 μg/ml DAPI (Sigma-Aldrich D9542), washed three times in PBS for 10 minutes, and once in deionized water before mounting them in VECTASHIELD Antifade Mounting Medium (Vector Laboratories H-1000).

#### Confocal Microscopy

Primary neurons, non-treated and treated with KCl and AP5, were imaged using a confocal microscope (Leica TCS SP5 on DM6000, equipped with MSD module) using an oil objective HCX PL APO CS 40.0X (NA = 1.25), and the pinhole was set to 1 Airy Unit (AU). The following laser lines/acquisition windows were used: Ar 514 nm / (520/540 nm) and Ar 543 nm / (650/700nm) for Venus and TdTomato, respectively. 1024x1024 images at digital zoom 5 were taken with 0.08 μm pixel size.

GluA1 immunolabelled neurons were imaged using Zeiss LSM 800 (oil objective - Plan-Apochromat 63×/1.4 NA DIC M27) confocal microscopes, and the pinhole was set to 1 Airy Unit (AU). The following laser lines were used: 640 nm, 561 nm and 488 nm for GluA1, TdTomato and mVenus, respectively. 1024x1024 images were taken with 0.099 μm pixel size.

Brain slice images (1024x1024 pixels) were acquired in the hippocampal area CA1 with a confocal microscope (Leica TCS SP5 on DM6000, equipped with MSD module) using an oil objective HCX PL APO CS 40.0X (NA=1.25), and the pinhole was set to 1 Airy Unit (AU). The area was identified by morphological criteria using the DAPI staining. Optical rotation was performed so that the CA1 pyramidal layer was parallel to one of the image axes, then the sample was moved so that the desired area would lie in the centre of the field of view. Digital zoom was set to 3, yielding a final pixel size of 0.13 μm. The following excitation wavelength/acquisition windows were used for the different fluorophores: DAPI (405 nm/415-465 nm) Alexa Fluor488 (488 nm/500-550 nm), Alexa Fluor555 (561 nm/565-650 nm), Alexa Fluor647 (633 nm/645,700 nm). Sequential illumination with HeNe 633, Ar 488, DPSSL 561 and diode (Picoquant, Berlin, Germany) 405 laser lines to minimize photobleaching.

#### ImageJ analysis

In cell culture experiments, the number of mVenus+ spines was counted and normalized by the total number of spines, counted from the tdTomato channel. AMPAR enrichment analysis was performed as previously described (Zhang et al., 2015). Briefly, circular ROIs of a radius approximately equal to the spine head (tdTomato+) were drawn over the spines with or without the SA-PSDΔVenus signal and background subtracted integrated AMPAR intensity was calculated for each ROI. Background intensities were obtained from the spine ROIs translated in x/y to a nearby region. The volume of a spine was calculated using the formula, 4/3 𝜋𝑟3, where *r* is the radius of the corresponding spine ROI.

In animal experiments, areas from different CA1 subfields were analyzed according to standard classification (Paxinos and Franklin, 1997; Andersen et al., 2007). *Stratum oriens* (SO) is defined as the tissue layer between the outer pyramidal layer and the surface of the hippocampus containing the basal dendrites; *stratum radiatum* (SR) is the layer containing the apical dendrites while the *stratum lacunosum-moleculare* (SLM) is the most distal part of CA1, comprising the last 50-60 μm before the hippocampal sulcus separating CA1 from the dentate gyrus. Morphologically, the SLM coincides with the apical tuft of CA1 pyramidal neurons (Spruston, 2008). To account for the larger area of SR compared to SO and SLM, SR was divided into a proximal part (pSR) closer to the soma layer and a distal part (dSR) closer to the SLM. Dendrites in the four sets were selected based on the mCherry channel, and then the number of SA-PSDΔVenus-positive spines was counted. The number is expressed as the number of SA-PSDΔVenus+ spines over the whole number of spines in the dendrite, as evaluated by the filler mCherry channel, normalized on the control group. Last, after counting, the dendrite was classified as ESARE-dTurquoise+ or ESARE-dTurquoise- based on the presence or absence of anti-FLAG staining in the 647 channel. Counting was performed blind to the experimental group.

### References

Gobbo, F., Marchetti, L., Jacob, A., Pinto, B., Binini, N., Pecoraro Bisogni, F., et al. (2017). Activity-dependent expression of Channelrhodopsin at neuronal synapses. *Nat Commun* 8, 1629. doi: 10.1038/s41467-017-01699-7

Lu, W., Man, H., Ju, W., Trimble, W. S., MacDonald, J. F., and Wang, Y. T. (2001). Activation of synaptic NMDA receptors induces membrane insertion of new AMPA receptors and LTP in cultured hippocampal neurons. *Neuron* 29, 243–54. doi: 10.1016/s0896-6273(01)00194-5
